# Supplementary material for: Efficient characterizations of multiphoton states with an ultra-thin optical device
Source: Nat Commun. 2024 May 10;15:3944. doi: 10.1038/s41467-024-48213-4 (PMC11087518; doi:10.1038/s41467-024-48213-4)
Supplement: Supplementary file 1 — Supplementary Information [file 41467_2024_48213_MOESM1_ESM.pdf]

Supplementary Information for: "Efficient Characterizations of Multiphoton States with an Ultra-thin Optical Device"

Kui An,[1,*] Zilei Liu,[2,3,*] Ting Zhang,[1] Siqi Li,[2] You Zhou,[4,5] Xiao Yuan,[6] Leiran Wang,[2,3] Wenfu Zhang,[2,3] Guoxi Wang,[2,3,†] and He Lu[1,7,‡]

[1] *School of Physics, State Key Laboratory of Crystal Materials, Shandong University, Jinan 250100, China.*
[2] *State Key Laboratory of Transient Optics and Photonics, Xi'an Institute of Optics and Precision Mechanics, Chinese Academy of Sciences, Xi'an 710119, China.*
[3] *University of Chinese Academy of Sciences, Beijing 100049, China.*
[4] *Key Laboratory for Information Science of Electromagnetic Waves (Ministry of Education), Fudan University, Shanghai 200433, China*
[5] *Hefei National Laboratory, Hefei 230088, China*
[6] *Center on Frontiers of Computing Studies, Peking University, Beijing 100871, China.*
[7] *Shenzhen Research Institute of Shandong University, Shenzhen 518057, China.*

SUPPLEMENTARY NOTE 1: SHADOW TOMOGRAPHY WITH POVM

A. Constructing classical shadow with POVM

Consider a d-level quantum system, a set of L rank-one projectors $\{|\psi_l\rangle\langle\psi_l| \in \mathbb{H}_d\}_{l=1}^L$ is called a quantum (complex projective) 2-design if the average value of second moment $(|\psi_l\rangle\langle\psi_l|)^{\otimes 2}$ over the set $\{|\psi_l\rangle\}$ is proportional to the projector onto the totally symmetric subspace of two copies

$$\frac{1}{L}\sum_{l=1}^{L}(|\psi_l\rangle\langle\psi_l|)^{\otimes 2} = \binom{d+1}{2}^{-1} P_{\mathrm{Sym}^{(2)}}, \tag{1}$$

where $P_{\mathrm{Sym}^{(2)}} = \frac{1}{2}(\mathbb{1}_d \otimes \mathbb{1}_d + \mathbb{F})$ is the projector of the symmetric subspace, and $\mathbb{F}$ is the swap operator acting on 2-copy as $\mathbb{F}|v\rangle \otimes |w\rangle = |w\rangle \otimes |v\rangle$ for all $|v\rangle, |w\rangle \in \mathbb{C}^d$.

Following the defining property Supplementary Eq. (1), each quantum 2-design is proportional to a POVM

$$\mathbf{E} = \{E_l = \frac{d}{L}|\psi_l\rangle\langle\psi_l|\}_{l=1}^{L}, \tag{2}$$

since the elements E_l are positive semidefinite and satisfy $\sum_{l=1}^{L} E_l = \mathbb{1}_d$. Measuring a quantum state ρ results in one of the L outcomes indexed by $l \in [L]$, and by Born's rule, the corresponding probability

$$p_l = \Pr(l|\rho) = \mathrm{Tr}(E_l\rho). \tag{3}$$

Hereafter we focus on the case of $d = 2$. The POVM defined in Supplementary Eq. (2) (together with the preparation

---

* These authors contributed equally to this work.
† wangguoxi@opt.ac.cn
‡ luhe@sdu.edu.cn

of the corresponding state $|\psi_l\rangle$) can be viewed as a linear map $\mathcal{M}:\mathbb{H}_2\to\mathbb{H}_2$ as follows

$$\begin{aligned}\mathcal{M}(\rho) &= \sum_{l=1}^{L}\Pr(l|\rho)\,|\psi_l\rangle\langle\psi_l| \\ &= \frac{2}{L}\sum_{l=1}^{L}\langle\psi_l|\,\rho\,|\psi_l\rangle\,|\psi_l\rangle\langle\psi_l| \\ &= \frac{2}{L}\mathrm{Tr}_1\left(\left(\sum_{l=1}^{L}(|\psi_l\rangle\langle\psi_l|)^{\otimes 2}\right)\mathbb{1}_2\otimes\rho\right) \\ &= 2\mathrm{Tr}_1\left(\frac{1}{6}(\mathbb{1}_2\otimes\mathbb{1}_2+\mathbb{F})\mathbb{1}_2\otimes\rho\right) \\ &= \frac{1}{3}\mathrm{Tr}_1\left(\mathbb{1}_2\otimes\rho+\rho\otimes\mathbb{1}_2\right) \\ &= \frac{1}{3}\left(\rho+\mathrm{Tr}(\rho)\mathbb{1}_2\right).\end{aligned} \tag{4}$$

The inverse of this map is

$$\mathcal{M}^{-1}(X) = 3X - \mathrm{Tr}(X)\mathbb{1}_2 \;\forall X\in\mathbb{H}_2. \tag{5}$$

For a single experimental run by performing POVM on ρ, we obtain the random outcome l with probability $\Pr(l|\rho)$, and the classical shadow is constructed according to Supplementary Eq. (5) as

$$\hat{\rho}_l = \mathcal{M}^{-1}(|\psi_l\rangle\langle\psi_l|) = 3\,|\psi_l\rangle\langle\psi_l| - \mathbb{1}_2. \tag{6}$$

It exactly reconstructs the underlying quantum state ρ in expectation

$$\begin{aligned}\mathbb{E}(\hat{\rho}_l) &= \sum_l \Pr(l|\rho)(3\,|\psi_l\rangle\langle\psi_l| - \mathbb{1}_2) \\ &= 3\sum_l \Pr(l|\rho)\,|\psi_l\rangle\langle\psi_l| - \mathbb{1}_2 \\ &= \rho+\mathbb{1}_2-\mathbb{1}_2 \\ &= \rho,\end{aligned} \tag{7}$$

where the third line is due to Supplementary Eq. (4).

For an N-qubit state $\rho\in\mathbb{H}_2^{\otimes N}$, the POVM $\mathbf{E}^{\otimes N}$ acts on each qubit independently yields outcome as a string $\boldsymbol{l}=l_1l_2\cdots l_N$ with probability

$$\begin{aligned}\Pr(\boldsymbol{l}|\rho) &= \mathrm{Tr}\left(\rho\bigotimes_{n=1}^{N}E_{l_n}\right) \\ &= \left(\frac{2}{L}\right)^N\langle\psi_{\boldsymbol{l}}|\,\rho\,|\psi_{\boldsymbol{l}}\rangle,\end{aligned} \tag{8}$$

where $|\psi_{\boldsymbol{l}}\rangle = |\psi_{l_1}\rangle\otimes|\psi_{l_2}\rangle\otimes\cdots\otimes|\psi_{l_N}\rangle$. For a single experimental run, the classical shadow shows

$$\begin{aligned}\hat{\rho} &= \bigotimes_{n=1}^{N}\mathcal{M}^{-1}(|\psi_{\boldsymbol{l}}\rangle\langle\psi_{\boldsymbol{l}}|) \\ &= \bigotimes_{n=1}^{N}\mathcal{M}^{-1}(|\psi_{l_n}\rangle\langle\psi_{l_n}|) \\ &= \bigotimes_{n=1}^{N}\left(3\,|\psi_{l_n}\rangle\langle\psi_{l_n}| - \mathbb{1}_2\right),\end{aligned} \tag{9}$$

which is in a tensor-product form.

B. Calibration of quantum channel $\mathcal{M}$

In practice, there are unavoidable noises in unitary operations and measurements. To address this issue, robust classical shadow (RShadow) protocol was proposed to mitigate the noise in shadow tomography [1]. It is convenient to represent $\mathcal{M}$ as a matrix $\mathcal{L}_{\mathcal{M}}$ in Pauli-Liouville representation. Here a linear operator X is represented by a column vector $|X\rangle\rangle_j = \mathrm{Tr}(\sigma_j X)$ in the Pauli-basis with $\sigma_0 = \mathbb{1}_2/\sqrt{2}$ and σ_1, σ_2, σ_3 being the Pauli matrix $X/\sqrt{2}$, $Y/\sqrt{2}$, $Z/\sqrt{2}$. In this way Supplementary Eq. (4) can be expressed as

$$\mathcal{L}_{\mathcal{M}} |\rho\rangle\rangle = \sum_l \langle\langle E_l | \rho\rangle\rangle |\psi_l\rangle\rangle = \frac{2}{L} \sum_l |\psi_l\rangle\rangle\langle\langle\psi_l| |\rho\rangle\rangle. \tag{10}$$

The matrix form $\mathcal{L}_{\mathcal{M}}$ of the channel $\mathcal{M}$ corresponds to the projector onto the subspace spanned by $\mathbf{E}$, which is given by

$$\mathcal{L}_{\mathcal{M}} = \frac{2}{L} \sum_{l=1}^{L} |\psi_l\rangle\rangle\langle\langle\psi_l| , \tag{11}$$

and the classical shadow is

$$|\hat{\rho}\rangle\rangle = \mathcal{L}_{\mathcal{M}}^{-1} |\psi_l\rangle\rangle, \tag{12}$$

where $\mathcal{L}_{\mathcal{M}}^{-1}$ is the Moore-Penrose pseudo inverse of $\mathcal{L}_{\mathcal{M}}$ [2].

The POVM $\mathbf{E}_{\mathrm{octa}} = \{\frac{1}{3} |\psi_l\rangle\langle\psi_l| : l = 1, \cdots, 6\}$ with corresponding normalized vector $|\psi_l\rangle \in \{|H\rangle, |V\rangle, |+\rangle, |-\rangle, |R\rangle, |L\rangle\}$. The matrix $\mathcal{L}_{\mathcal{M}}$ and its inverse $\mathcal{L}_{\mathcal{M}}^{-1}$ are

$$\mathcal{L}_{\mathcal{M}} = \begin{pmatrix} 1 & 0 & 0 & 0 \\ 0 & \frac{1}{3} & 0 & 0 \\ 0 & 0 & \frac{1}{3} & 0 \\ 0 & 0 & 0 & \frac{1}{3} \end{pmatrix}, \quad \mathcal{L}_{\mathcal{M}}^{-1} = \begin{pmatrix} 1 & 0 & 0 & 0 \\ 0 & 3 & 0 & 0 \\ 0 & 0 & 3 & 0 \\ 0 & 0 & 0 & 3 \end{pmatrix}. \tag{13}$$

Accordingly, one has

$$\mathcal{L}_{\mathcal{M}} |\rho\rangle\rangle = \frac{1}{3}(|\rho\rangle\rangle + |\mathbb{1}_2\rangle\rangle), \tag{14}$$

which is a depolarizing channel. Accordingly, the classical shadow in Supplementary Eq. (6) can be written as

$$|\hat{\rho}\rangle\rangle = \mathcal{L}_{\mathcal{M}}^{-1} |\psi_l\rangle\rangle = 3 |\psi_l\rangle\rangle - |\mathbb{1}_2\rangle\rangle \Leftrightarrow \hat{\rho} = 3 |\psi_l\rangle\langle\psi_l| - \mathbb{1}_2. \tag{15}$$

Similarly, for an N-qubit state ρ,

$$\begin{aligned} \mathcal{L}_{\mathcal{M}} |\rho\rangle\rangle &= \sum_{\boldsymbol{l}} \mathrm{Pr}(\boldsymbol{l}|\rho) |\psi_{\boldsymbol{l}}\rangle\rangle \\ &= \sum_{l_1, l_2, \cdots, l_N} \langle\langle \bigotimes_{n=1}^{N} E_{l_n} | \rho\rangle\rangle | \bigotimes_{n=1}^{N} \psi_{l_n}\rangle\rangle \\ &= \bigotimes_{n=1}^{N} \left[\frac{1}{3} \sum_{l_n} |\psi_{l_n}\rangle\rangle\langle\langle\psi_{l_n}| \right] |\rho\rangle\rangle \\ &= \bigotimes_{n=1}^{N} \mathcal{L}_{\mathcal{M}_n} |\rho\rangle\rangle. \end{aligned} \tag{16}$$

Then we have

$$\mathcal{L}_{\mathcal{M}} = \bigotimes_{n=1}^{N} \mathcal{L}_{\mathcal{M}_n}, \tag{17}$$

and the classical shadow is

$$
\begin{aligned}
|\hat{\rho}\rangle\rangle &= \mathcal{L}_{\mathcal{M}}^{-1}\bigotimes_{n=1}^{N}|\psi_{l_n}\rangle\rangle \\
&= \bigotimes_{n=1}^{N}\mathcal{L}_{\mathcal{M}_n}^{-1}|\psi_{l_n}\rangle\rangle \\
&= \bigotimes_{n=1}^{N}(3|\psi_{l_n}\rangle\rangle - |\mathbb{1}_2\rangle\rangle).
\end{aligned} \tag{18}
$$

The quantum channel in Supplementary Eq. (17) can be written as

$$
\mathcal{L}_{\mathcal{M}} = \sum_{\lambda\in\{0,1\}^N} f_\lambda \Pi_\lambda, \tag{19}
$$

where λ is an n-bit vector denoting the subspaces due to the irreducible representation, and $\Pi_\lambda = \bigotimes_{n=1}^{N}\Pi_{\lambda_n}$ in the tensor-product form with

$$
\Pi_{\lambda_n} = \begin{cases} |\sigma_0\rangle\rangle\langle\langle\sigma_0|, & \lambda_n = 0 \\ \mathbb{1}_4 - |\sigma_0\rangle\rangle\langle\langle\sigma_0|, & \lambda_n = 1. \end{cases} \tag{20}
$$

Here $\sigma_0 = \mathbb{1}_2/\sqrt{2}$ is the normalized single-qubit identity operator such that $\langle\langle\sigma_0|\,\sigma_0\rangle\rangle = 1$, and $\mathbb{1}_4$ is the 4-dimensional identity matrix for this single-qubit operator space. That is, Π_0 subspace corresponds to the identity operator, and Π_1 is the complementary subspace spanned by the Pauli operators evenly. In the noiseless case,

$$
f_\lambda = \frac{1}{3^{|\lambda|}}, \tag{21}
$$

where $|\lambda|$ is the number of 1s in the n-bit vector λ. For the single-qubit case with $N = 1$, the matrix form of $\mathcal{L}_{\mathcal{M}}$ is just shown in Supplementary Eq. (13).

Considering the noise (or imperfections) in practice, suppose the corresponding quantum channel of the noisy POVM can be written as $\mathcal{L}_{\widetilde{\mathcal{M}}} = \sum_\lambda \tilde{f}_\lambda \Pi_\lambda$, which is still diagonal according to the subspaces. The noisy parameters $\tilde{f}_\lambda$ can be experimentally calibrated.

To this end, a high-fidelity N-qubit product state

$$
\rho_{\mathbf{0}} = \bigotimes_{n=1}^{N}|0\rangle\langle 0| \Leftrightarrow |\rho_{\mathbf{0}}\rangle\rangle = \bigotimes_{n=1}^{N}\frac{1}{2}(|\mathbb{1}_2\rangle\rangle + |Z\rangle\rangle), \tag{22}
$$

is prepared and measured with noisy POVM, and the probability is $\tilde{\Pr}(\boldsymbol{l}) = \langle\langle\tilde{E}_{\boldsymbol{l}}|\,\rho_{\mathbf{0}}\rangle\rangle$ with $|\tilde{E}_{\boldsymbol{l}}\rangle\rangle$ being the noisy POVM.

Here we define $|P_\lambda\rangle\rangle = \bigotimes_{n=1}^{N}|Z^{\lambda_n}\rangle\rangle$, we can use $\langle\langle P_\lambda|\,\psi_{\boldsymbol{l}}\rangle\rangle$ to estimate the noisy parameter $\tilde{f}_\lambda$. To show this is indeed an unbiased estimator, we take the expectation value and find that

$$
\begin{aligned}
\mathbb{E}\langle\langle P_\lambda|\,\psi_{\boldsymbol{l}}\rangle\rangle =& \sum_{\boldsymbol{l}}\langle\langle P_\lambda|\,\psi_{\boldsymbol{l}}\rangle\rangle\langle\langle\tilde{E}_{\boldsymbol{l}}|\,\rho_{\mathbf{0}}\rangle\rangle \\
=& \langle\langle P_\lambda|\,\mathcal{L}_{\widetilde{\mathcal{M}}}\,|\rho_{\mathbf{0}}\rangle\rangle \\
=& \frac{1}{2^N}\left(\bigotimes_{n=1}^{N}\langle\langle Z^{\lambda_n}|\right)\sum_{\lambda'}\tilde{f}_{\lambda'}\Pi_{\lambda'}\bigotimes_{n=1}^{N}(|\mathbb{1}_2\rangle\rangle + |Z\rangle\rangle) \\
=& \frac{1}{2^N}\left(\bigotimes_{n=1}^{N}\langle\langle Z^{\lambda_n}|\right)\sum_{\lambda'}\tilde{f}_{\lambda'}\bigotimes_{n=1}^{N}|Z^{\lambda'_n}\rangle\rangle = \tilde{f}_\lambda.
\end{aligned} \tag{23}
$$

Experimentally, we record the results of $\boldsymbol{l}$ from the noisy POVM, and then calculate the value $\langle\langle P_\lambda|\,\psi_{\boldsymbol{l}}\rangle\rangle$ on the classical computer which serves as an estimation of $\tilde{f}_\lambda$. One can repeat this process M' times, and average them to

make the estimation more accurate. In this way, we can get the noisy parameters $\tilde{f}_\lambda$ of the noisy channel $\widetilde{\mathcal{M}}$, and the matrix form of its inverse $\widetilde{\mathcal{M}}^{-1}$ is

$$\mathcal{L}_{\widetilde{\mathcal{M}}}^{-1} = \sum_{\lambda \in \{0,1\}^N} \tilde{f}_\lambda^{-1} \Pi_\lambda. \tag{24}$$

In post-processing, we can use this new inverse channel $\widetilde{\mathcal{M}}$ instead of $\mathcal{M}$, such that the error in the noisy POVM could be mitigated.

C. Gate-dependent noise

In Ref. [1], two main assumptions are made on the noise of measurement device to make the theoretical framework rigorous and analytical, i.e.,

A1. The noise in the circuit is gate-independent, time-stationary, Markovian noise.

A2. The experimental device can generate the computational basis state $|\mathbf{0}\rangle \equiv |0\rangle^{\otimes N}$ with sufficiently high fidelity.

Our experimental device is able to generate $|HH\rangle$ with high fidelity (> 0.99) so that **A2** is satisfied. Metasurface is a passive optical device so that the metasurface-induced noise is time-stationary and Markovian noise as well. Note that measurement errors induced by metasurface are not strictly gate-independent, as the errors in σ_x, σ_y, and σ_z measurements are 0.086 ± 0.005, 0.073 ± 0.005, and 0.101 ± 0.005, respectively. However, it has been shown robust shadow tomography still works for gate-dependent noise [1]. Our experimental results (Figure 4 in main text) confirm this claim as well. To further confirm this point, we simulate the SLST and robust SLST on single-qubit state

$$\rho' = \left(1 - \frac{\delta_x + \delta_y + \delta_z}{3}\right)\rho + \frac{\delta_x}{3}\sigma_x\rho\sigma_x + \frac{\delta_y}{3}\sigma_y\rho\sigma_y + \frac{\delta_z}{3}\sigma_z\rho\sigma_z. \tag{25}$$

In each single run in simulation, the value of $\delta_{x,y,z}$ is randomly resampled from Gaussian distribution with mean value of $\bar{\delta}$ and standard deviation of σ. The simulation is equivalent to performing measurement with gate-dependent noises on the ideal state ρ. As shown in Supplementary Fig. 1 (a), the enhancement of robust SLST is not obvious when the noise is weak ($\bar{\delta} = 0.05$). Robust SLST exhibits advantage when the noise is strong ($\bar{\delta} = 0.1$) as shown in Supplementary Fig. 1 (b). The simulation results agree well with the experimental results shown in Figure 4 in main text, where the robust SLST significantly enhances the accuracy when optical loss is high.

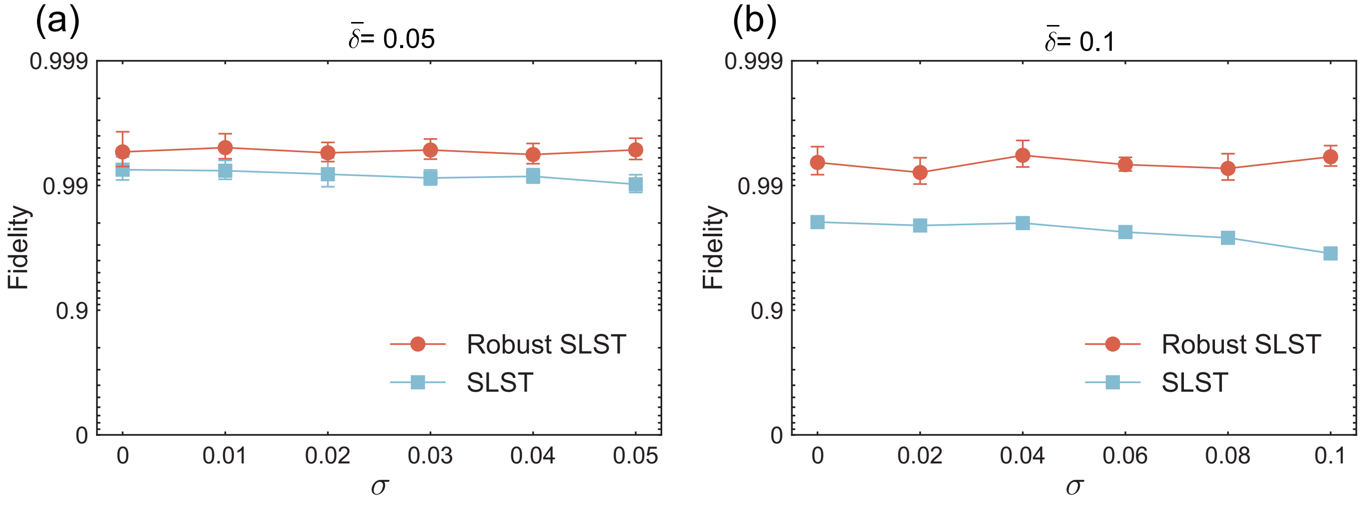

Supplementary Fig 1. **The simulation of robust SLST and SLST on the state in Supplementary Eq.** (25)**.** (a), The simulation is performed by setting $\bar{\delta} = 0.05$ and $\sigma \in [0, 0.05]$ with interval of 0.01. (b), The simulation is performed by setting $\bar{\delta} = 0.1$ and $\sigma \in [0, 0.1]$ with interval of 0.02. The simulation is carried out with $M = 2000$ runs. In robust SLST, additional $M' = 2000$ runs are used for calibration. We set $k = 100$, hyperparameters $a_1 = 34.1$ and $b_1 = 5.7$. Error bars represent standard deviations obtained by repeating the simulation 5 times.

D. Shadow norm

For a given POVM $\mathbf{E}$, the shadow norm of observable O is derived from the variance of the estimation $\hat{o}$ on state ρ. The variance $\mathrm{Var}(\hat{o})$ can be ideally written as

$$\mathrm{Var}(\hat{o}) = \sum_{l=1}^{L} \mathrm{Tr}(\hat{\rho}_l O)^2 \mathrm{Tr}(\rho E_l) - \mathrm{Tr}(\rho O)^2. \tag{26}$$

The shadow norm is then defined by the maximization of variance $\mathrm{Var}(\hat{o})$ over ρ

$$\mathrm{Var}(\hat{o}) = \sum_{l=1}^{L} \mathrm{Tr}(\hat{\rho}_l O)^2 \mathrm{Tr}(\rho E_l) - \mathrm{Tr}(\rho O)^2 \leq \max_{\rho} \sum_{l=1}^{L} \mathrm{Tr}(\hat{\rho}_l O)^2 \mathrm{Tr}(\rho E_l) - \mathrm{Tr}(\rho O)^2. \tag{27}$$

Note that the second term $\mathrm{Tr}(\rho O)^2$ is a constant and can be ignored. Then, the shadow norm of O is calculated by

$$||O||_{\mathrm{shd}}^2 = \lambda_{\max} \left\{ \sum_{l=1}^{L} \mathrm{Tr}(\hat{\rho}_l O)^2 E_l \right\}, \tag{28}$$

with $\lambda_{\max}\{\cdot\}$ being the maximal eigenvalue of corresponding operator. In theoretical investigations such as [3], it is convenient to calculate shadow norm in Supplementary Eq. (28). For octahedron POVM, the theoretical shadow norm calculated according to Supplementary Eq. (28) is $||O||_{\mathrm{shd}}^2 = 1.5$.

Experimentally, the variance of estimator $\hat{o}$ is observed by

$$\mathrm{Var}(\hat{o}) = \frac{1}{M} \sum_{m=1}^{M} \left(\hat{o}^{(m)} - \hat{O} \right)^2. \tag{29}$$

Without consideration of experimental noise, Supplementary Eq. (29) converges to Supplementary Eq. (26) when $M \to \infty$. For octahedron POVM, the maximization of Supplementary Eq. (26) over single-qubit pure state ρ yields $||O||_{\mathrm{shd}}^2 = 0.75$, which is considered as the ideal value for experimentally observed maximal variance in Supplementary Eq. (29).

E. Simulation of shadow norm with SIC POVM

The SIC POVM in qubit system can be expressed by $\mathbf{E}_{\mathrm{SIC}} = \{\frac{1}{2} |\psi_l\rangle \langle\psi_l|\}_{l=1}^{4}$ with

$$\begin{aligned} |\psi_1\rangle &= |0\rangle \\ |\psi_2\rangle &= \frac{1}{\sqrt{3}}|0\rangle + \sqrt{\frac{2}{3}}|1\rangle \\ |\psi_3\rangle &= \frac{1}{\sqrt{3}}|0\rangle + \sqrt{\frac{2}{3}}e^{i\frac{2\pi}{3}}|1\rangle \\ |\psi_4\rangle &= \frac{1}{\sqrt{3}}|0\rangle + \sqrt{\frac{2}{3}}e^{i\frac{4\pi}{3}}|1\rangle. \end{aligned} \tag{30}$$

For fixed observable $O = |\psi_{\kappa,\nu}\rangle \langle\psi_{\kappa,\nu}| \in \mathbf{O}$, single-qubit state $\rho = |\psi_{\gamma,\phi}\rangle \langle\psi_{\gamma,\phi}|$ and $\mathbf{E}_{\mathrm{SIC}}$, we simulate the statistic of outcomes with M runs, and calculate the variance according to Supplementary Eq. (29). Then, the shadow norm is calculated by maximization over state set $\mathbf{P}$, i.e., $\max_{\mathbf{P}} \mathrm{Var}(\hat{o}^{(m)})$, where $\mathbf{P}$ is the set of 20 pure states.

SUPPLEMENTARY NOTE 2: DETAILS OF SLST

A. Loss function in SLST

Generally, the loss function in SLST is squared Frobenius norm between two matrices τ and ρ defined as

$$N_F(\tau, \rho) = \|\rho - \tau\|_F^2 = \mathrm{Tr}(\rho - \tau)^2 = \mathrm{Tr}(\rho^2) + \mathrm{Tr}(\tau^2) - 2\mathrm{Tr}(\rho\tau). \tag{31}$$

For this loss function, we can write down the unbiased estimator with shadows $\{\hat{\rho}^{(m)}\}_{m=1}^{M}$ as follows. For the first term, it shows

$$\frac{2}{M(M-1)}\sum_{m<n}\mathrm{Tr}\left[\hat{\rho}^{(m)}\hat{\rho}^{(n)}\right], \tag{32}$$

which is an order-2 polynomial function of $\hat{\rho}$ [4]. Obviously, $\mathrm{Tr}(\hat{\rho}\tau)$ is an unbiased estimator as well. Then, the unbiased estimator of N_F in total is

$$\hat{N}_F(\tau) = \frac{2}{M(M-1)}\sum_{m<n}\mathrm{Tr}\left[\hat{\rho}^{(m)}\hat{\rho}^{(n)}\right] + \mathrm{Tr}(\tau^2) - 2\sum_{m}\mathrm{Tr}(\hat{\rho}^{(m)}\tau). \tag{33}$$

Accordingly, the gradient is

$$\boldsymbol{g}_k = \frac{\hat{N}_F(\boldsymbol{r}_k + B_k\boldsymbol{\Delta}_k) - \hat{N}_F(\boldsymbol{r}_k - B_k\boldsymbol{\Delta}_k)}{2B_k}\boldsymbol{\Delta}_k. \tag{34}$$

Note that fidelity F can also be used as loss function if τ is pure state, as $\hat{F}(\tau)$ is an unbiased estimation with classical shadows $\{\hat{\rho}^{(m)}\}$

$$\hat{F}(\tau) = \sum_{m}\mathrm{Tr}\left(\hat{\rho}^{(m)}\tau\right), \tag{35}$$

and the gradient is

$$\boldsymbol{g}_k = \frac{\hat{F}(\boldsymbol{r}_k + B_k\boldsymbol{\Delta}_k) - \hat{F}(\boldsymbol{r}_k - B_k\boldsymbol{\Delta}_k)}{2B_k}\boldsymbol{\Delta}_k. \tag{36}$$

B. Setting of hyperparameters in SPSA optimization

The setting of hyperparameters a_1, a_2, a_3, b_1 and b_2 determines the convergence of SPSA optimization. Previous investigations have concluded that $a_3 = 0.602, b_2 = 0.101$ are generally good choices for most optimization tasks [5–7], so that we set $a_3 = 0.602, b_2 = 0.101$ in our optimization. Besides, we find that a_2 is trivial compared with other four hyperparameters so that we set $a_2 = 0$. a_1 and b_1 are determined through numerical simulations. We set $a_1 = 13$, $b_1 = 0.5$ in single-photon experiment, while $a_1 = 8.5$ and $b_1 = 1.4$ in two-photon experiment.

C. Model of proposed state τ_k in SLST

For proposed state τ_k being an N-qubit pure state $\tau_k = |\zeta\rangle\langle\zeta|$ with dimension $d = 2^N$, it can be formalized by

$$|\zeta\rangle = \frac{1}{\sqrt{\sum_{i=1}^{d} r_i^2}}\begin{pmatrix} r_1 \\ r_2 e^{ir_{d+1}} \\ r_3 e^{ir_{d+2}} \\ \vdots \\ r_d e^{ir_{2d-1}} \end{pmatrix}, \tag{37}$$

and we set $\boldsymbol{r}_k = \{r_1, r_2, \cdots, r_{2d-1}\}$ with $r_i \in \mathbb{R}$ in SPSA optimization.

For general case, a mixed state, the proposed state τ_k is modeled by Cholesky decomposition

$$\tau_k = \frac{TT^\dagger}{\mathrm{Tr}(TT^\dagger)}, \tag{38}$$

where T is a lower triangular matrix

$$T = \begin{pmatrix} r_1 & 0 & \cdots & 0 \\ r_{d+1} + ir_{d+2} & r_2 & \cdots & 0 \\ \vdots & \vdots & \ddots & \vdots \\ r_{d^2-1} + ir_{d^2} & r_{d^2-3} + ir_{d^2-2} & \cdots & r_d \end{pmatrix}. \tag{39}$$

Accordingly, we set $\boldsymbol{r}_k = \{r_1, r_2, \cdots, r_{d^2}\}, r_i \in \mathbb{R}$ in SPSA optimization.

D. Scaling of SLST

To investigate the scaling of SLST, we simulate SLST with POVM $\mathbf{E}_{\rm octa}$ on 50 randomly generated N-qubit pure states ρ_N with $N = 2, 4, 6$ and 8, respectively. The results of infidelity $1 - F(\tau_k, \rho_N)$ are shown in Supplementary Fig. 2, in which we set the iterations $k = 200, 1000, 5000$ and 25000 for $N = 2$, 4, 6 and 8, respectively. The extracted scaling of SLST is $O(d \log d/M)$, which is slightly worse than $O(d/M)$ in SQT and $O(d^\eta/M)(\eta > 1)$ in SGQT. However, SLST with POVM requires only one experimental setting and the POVM is locally implemented on individual qubit, which is friendly to experiment. A comparison of these technologies is shown in Table 1.

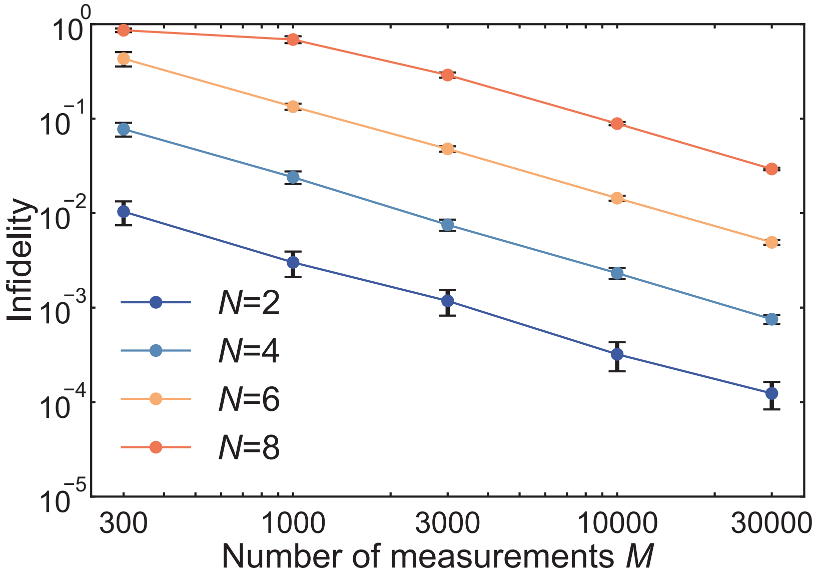

Supplementary Fig 2. **The average infidelity of 50 reconstructed states with number of measurements** M**.** The hyperparameters we set in SPSA optimization are $a_1 = 20, b_1 = 0.35$ for $N = 2$, $a_1 = 15, b_1 = 0.79$ for $N = 4$, $a_1 = 30, b_1 = 0.92$ for $N = 6$ and $a_1 = 77, b_1 = 0.92$ for $N = 8$. The error bars are the standard deviations of infidelity over 50 states.

	Infidelity	Experimental Setting	Measurement Type	Online/Offline
SQT	$O(d/M)$	$O(d)$	Global projective measurement	Offline
SGQT	$O(d^\eta/M)$	$O(M)$	Global projective measurement	Online
SLST with POVM	$O(d \log d/M)$	1	Local POVM	Online

Supplementary Table 1. Comparison of SQT, SGQT and SLST with POVM.

E. Initial setting of τ_0

Generally, achieving the global minimum instead of local minimum is challenging in optimization. Indeed, SPSA optimization avoids local minimum under asymptotic iterations due to stochastic perturbation [7]. However, SPSA optimization does not guarantee the global convergence in each iteration [6, 7]. We show that the global convergence in each iteration can be improved by setting initial τ_0 with prior information, instead of randomly setting initial τ_0.

The direct estimation from classical shadows $\hat{\rho} = \frac{1}{M}\sum_{m=1}^{M} \hat{\rho}^{(m)}$ returns a Hermitian matrix, which has an eigen-decomposition in form of

$$\hat{\rho} = \sum_{i=1}^{d} \lambda_i \left|\Psi_i\right\rangle \left\langle\Psi_i\right|, \tag{40}$$

with λ_i and $|\Psi_i\rangle$ being the eigenvalue and eigenvector of $\hat{\rho}$. However, $\hat{\rho}$ is not a semi-positive matrix so that λ_i might

be negative. We set the initial τ_0 as

$$\tau_0 = \frac{\sum_{i=1}^{d} |\lambda_i| \left|\Psi_i\right\rangle \left\langle\Psi_i\right|}{\sum_{i=1}^{d} |\lambda_i|}, \tag{41}$$

which is close to $\hat{\rho}$. Then, we do Cholesky decomposition on τ_0 according to Supplementary Eq. (38), and obtain the corresponding $\boldsymbol{r}_0$ to start SPSA optimization.

To show the advantage of this modification, we simulate the SLST with $M = 2000, 3500$ and 5000 runs on randomly generated 2-, 3- and 4-qubit mixed states, respectively. The results are shown in Supplementary Fig. 3. We observe that the initial setting of τ_0 significantly influences efficiency and accuracy of SLST. With modified τ_0, SLST converges more quickly and achieves lower infidelity than that with random setting of τ_0.

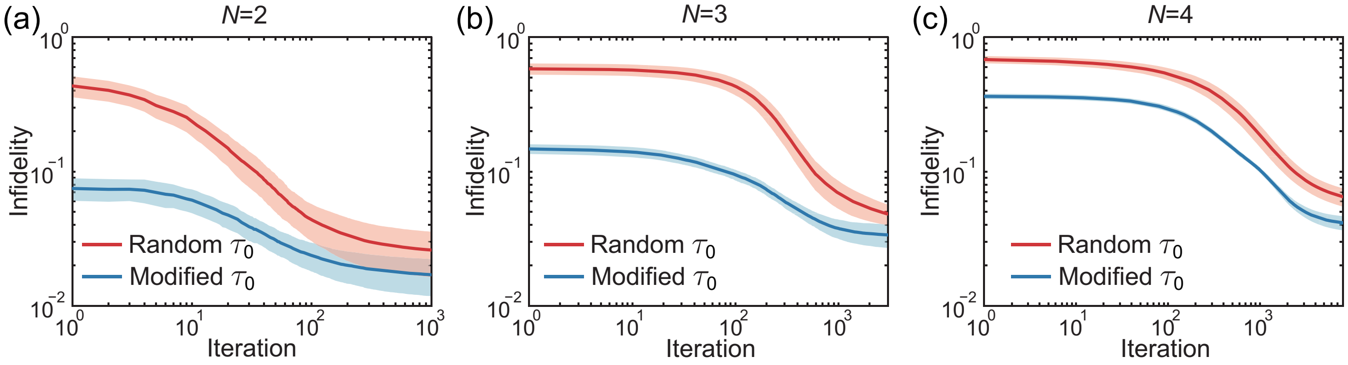

Supplementary Fig 3. Simulation of SLST with different initial τ_0. Blue line and red line represent the infidelity against iteration with modified τ_0 and random τ_0 of SLST on **(a)** $N = 2$, **(b)** $N = 3$ and **(c)** $N = 4$ mixed states, respectively. The hyperparameters we set in SPSA with modified τ_0 are $a_1 = 4.3, b_1 = 0.5$ for $N = 2$, $a_1 = 6.2, b_1 = 1.4$ for $N = 3$ and $a_1 = 16.4, b_1 = 2.8$ for $N = 4$. The hyperparameters we set in SPSA with random τ_0 are $a_1 = 48, b_1 = 1.1$ for $N = 2$, $a_1 = 44, b_1 = 8.0$ for $N = 3$ and $a_1 = 49, b_1 = 12.7$ for $N = 4$. The shadings represent the standard deviation of infidelities over 100 randomly generated mixed states.

SUPPLEMENTARY NOTE 3: EXPERIMENTAL DEMONSTRATION OF SGQT ON SINGLE-PHOTON STATE WITH METASURFACE

Self-guided quantum tomography (SGQT) is an iterative protocol to reconstruct underlying state of ρ. In fact, it is a SPSA optimization of the problem

$$\begin{aligned} &\text{maximize} && F(\tau^{\text{SGQT}}) = \sqrt{\text{Tr}(\rho\tau^{\text{SGQT}})} \\ &\text{subject to} && \tau^{\text{SGQT}} \geq 0,\ \text{Tr}(\tau^{\text{SGQT}}) = 1, \end{aligned} \tag{42}$$

where τ^{SGQT} is the proposed state that is positive semidefinite ($\tau \geq 0$) with unit trace ($\text{Tr}(\tau^{\text{SGQT}}) = 1$), and the loss function is the squared state fidelity $F(\tau^{\text{SGQT}})$. Note that τ^{SGQT} is restricted to pure states in SGQT. As an $N-$qubit pure state τ^{SGQT} can be modeled with $2d-1$ parameters, so that we denote τ^{SGQT} by a $2d$-dimensional vector $\boldsymbol{r} = [r_1, r_2, \cdots, r_{2d}]$. Accordingly, squared state fidelity is denoted by $F(\boldsymbol{r}) = F(\rho, \tau)$, which equals to the probability of projection ρ on τ^{SGQT}.

The process of SGQT algorithm can be described as follows.

1. Randomly guessing an initial state τ_0^{SGQT};

2. Perturb $\tau_0(\boldsymbol{r})$ with a random perturbation vector $\boldsymbol{\Delta}_k = [\Delta_{k1}, \Delta_{k2}, \cdots, \Delta_{kd^2}]$ with Δ_{ki} being generated from Bernoulli ± 1 distribution with equal probability;

3. Implement projective measurement on perturbed states $\tau(\boldsymbol{r}_k + B_k\boldsymbol{\Delta}_k)$ and $\tau(\boldsymbol{r}_k - B_k\boldsymbol{\Delta}_k)$;

4. Calculate the gradient by

$$\boldsymbol{g}_k = \frac{F(\boldsymbol{r}_k + B_k\boldsymbol{\Delta}_k) - F(\boldsymbol{r}_k - B_k\boldsymbol{\Delta}_k)}{2B_k}\boldsymbol{\Delta}_k, \tag{43}$$

and update $\boldsymbol{r}_k$ to $\boldsymbol{r}_{k+1} = \boldsymbol{r}_k + A_k \boldsymbol{g}_k$;

5. Repeat step 2-step 4 until $\boldsymbol{g}_k$ converges to zero, and corresponding τ_k is the reconstructed state with SGQT protocol.

Experimentally, we demonstrate SGQT on single-photon state $|\psi_{\gamma,\phi}\rangle \in \mathbf{P}$ with an E-QWP, an E-HWP and metasurface. Note that only the photon passing through region 1 of the metasurface is post-selected in SGQT, which acts as a PBS. The projective measurement on τ^{SGQT} and its orthogonal state $\tau_{\perp}^{\text{SGQT}}$ is realized by setting the angles of E-QWP and E-HWP. Consequently, the region 1 of metasurface deflects τ^{SGQT} and $\tau_{\perp}^{\text{SGQT}}$ into opposite directions. By collecting the counts at two directions, we can calculate the probability of projection on τ^{SGQT} and $\tau_{\perp}^{\text{SGQT}}$, respectively. In each iteration, seven experimental runs are carried out to project $|\psi_{\gamma,\phi}\rangle$ on $\tau_{k+}^{\text{SGQT}} = \boldsymbol{r}_k + B_k \boldsymbol{\Delta}_k$ and $\tau_{k-}^{\text{SGQT}} = \boldsymbol{r}_k - B_k \boldsymbol{\Delta}_k$, i.e., four experimental runs for $\tau_{k+}^{\text{SGQT}} = \boldsymbol{r}_k + B_k \boldsymbol{\Delta}_k$ and three experimental runs for $\tau_{k-}^{\text{SGQT}} = \boldsymbol{r}_k - B_k \boldsymbol{\Delta}_k$, respectively.

SUPPLEMENTARY NOTE 4: DESIGN, FABRICATION AND CHARACTERIZATION OF METASURFACE

A. Design of metasurface to realize POVM $\mathbf{E}_{\text{octa}}$

The POVM $\mathbf{E}_{\text{octa}}$ is equivalent to randomly selecting three Pauli observables and then performing projective measurement on its eigenstates. To this end, the metasurface is designed to consist of three regions with same size ($210\mu m \times 70\mu m$), each of which corresponds to the projective measurement of observable $\sigma_j, j \in \{x, y, z\}$. Each region is further designed to spatially separate eigenstates $|\psi^+_{\sigma_j}\rangle$ and $|\psi^-_{\sigma_j}\rangle$ of σ_j, which is achieved by individual phase control of $|\psi^+_{\sigma_j}\rangle$ and $|\psi^-_{\sigma_j}\rangle$ when they pass through metasurface by

$$\Phi^{\pm}_{\sigma_j}(x,y) = -\frac{2\pi}{\lambda}\left(\sqrt{(x - x^{\pm}_{\sigma_j,0})^2 + (y - y^{\pm}_{\sigma_j,0})^2 + f^2} - f\right). \tag{44}$$

$\Phi^{\pm}_{\sigma_j}(x,y)$ represents phase configuration at the output of metasurface with input polarization $|\psi^+_{\sigma_j}\rangle$ and $|\psi^-_{\sigma_j}\rangle$, $(x^+_{\sigma_j,0}, y^+_{\sigma_j,0})$ and $(x^-_{\sigma_j,0}, y^-_{\sigma_j,0})$ are the positions of separated focal spots of $|\psi^+_{\sigma_j}\rangle$ and $|\psi^-_{\sigma_j}\rangle$ at focal plane and f is the focal length. Our aim is to design a metasurface to realize phase configuration of $\Phi^{\pm}_{j}(x,y)$ calculated according to Supplementary Eq. (44) with fixed $(x^{\pm}_{\sigma_j,0}, y^{\pm}_{\sigma_j,0})$, λ and f. The parameters we set to calculate $\Phi^{\pm}_{\sigma_j}(x,y)$ are shown in Table 2.

Parameters	Values
x in $\Phi^{\pm}_{\sigma_x}(x,y)$	$[-105\mu\text{m}, 105\mu\text{m}]$ with interval of 0.5μm
y in $\Phi^{\pm}_{\sigma_x}(x,y)$	$[-35\mu\text{m}, 35\mu\text{m}]$ with interval of 0.5μm
$(x^{\pm}_{\sigma_x,0}, y^{\pm}_{\sigma_x,0})$	$(\pm 35\mu\text{m}, 0\mu\text{m})$
x in $\Phi^{\pm}_{\sigma_y}(x,y)$	$[-105\mu\text{m}, 105\mu\text{m}]$ with interval of 0.5μm
y in $\Phi^{\pm}_{\sigma_y}(x,y)$	$[-105\mu\text{m}, -35\mu\text{m}]$ with interval of 0.5μm
$(x^{\pm}_{\sigma_y,0}, y^{\pm}_{\sigma_y,0})$	$(\pm 35\mu\text{m}, -70\mu\text{m})$
x in $\Phi^{\pm}_{\sigma_z}(x,y)$	$[-105\mu\text{m}, 105\mu\text{m}]$ with interval of 0.5μm
y in $\Phi^{\pm}_{\sigma_z}(x,y)$	$[35\mu\text{m}, 105\mu\text{m}]$ with interval of 0.5μm
$(x^{\pm}_{\sigma_z,0}, y^{\pm}_{\sigma_z,0})$	$(\pm 35\mu\text{m}, 70\mu\text{m})$
f	150μm
λ	810nm

Supplementary Table 2. The values of parameters set in calculation of phase configurations $\Phi^{\pm}_{\sigma_j}(x,y)$ in Supplementary Eq. (44).

The calculated phase configurations of $\Phi^{+}_{\sigma_j}(x,y)$ and $\Phi^{-}_{\sigma_j}(x,y)$ are shown in Supplementary Fig. 4(b). To realize phase configurations $\Phi^{\pm}_{\sigma_j}(x,y)$, we design the metasurface consisting of 420×140 nanopillars with interval of 500nm. As shown in Supplementary Fig. 4(a), the nanopillar located at position (x_i, y_i) can be regarded as a polarization-dependent scatter acting on input polarization with transformation matrix

$$U_{\text{pillar}} = \begin{pmatrix} \cos\theta & -\sin\theta \\ \sin\theta & \cos\theta \end{pmatrix} \begin{pmatrix} e^{i\varphi_{x'}} & 0 \\ 0 & e^{i\varphi_{y'}} \end{pmatrix} \begin{pmatrix} \cos\theta & \sin\theta \\ -\sin\theta & \cos\theta \end{pmatrix}. \tag{45}$$

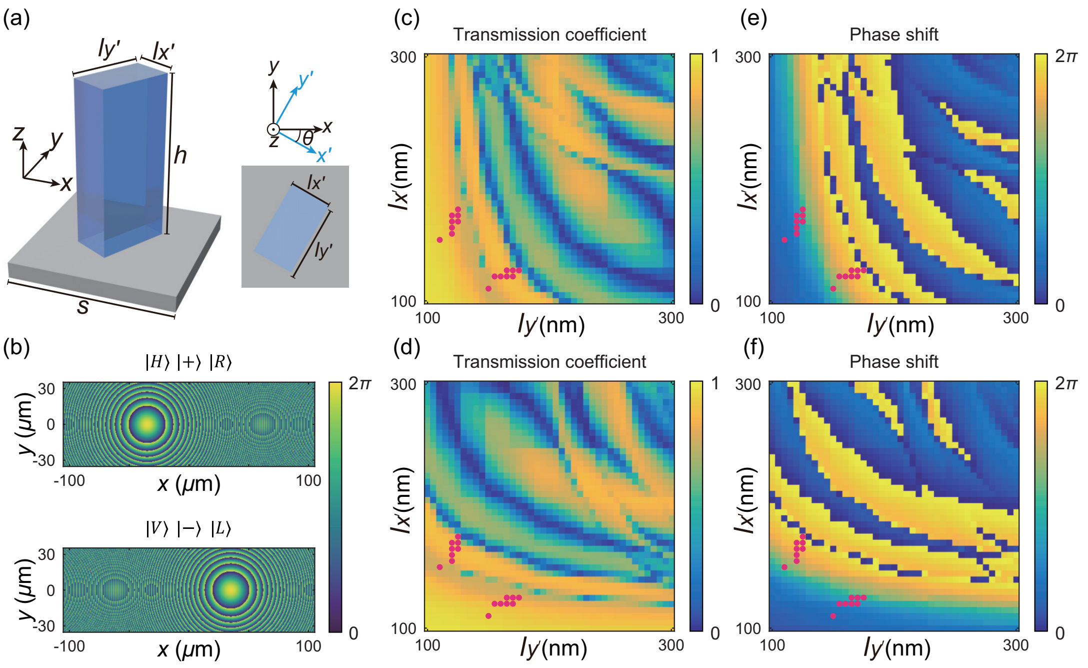

Supplementary Fig 4. **Schematic diagram and design of the metasurface. (a),** Schematic of the designed meta-atom consisting of an a-Si nanopillar on a fused-silica substrate. **(b),** Target phase configuration for orthogonally polarized states when light passes through metasurface. **(c)** and **(d),** Mapping of transmission coefficient for LP light polarized along x' and y', respectively, as a function of the parameters of $l_{x'}$ and $l_{y'}$ of the nanopillars. **(e)** and **(f),** Mapping of phase shift $\varphi_{x'}$ and $\varphi_{y'}$, respectively, as a function of the parameters of $l_{x'}$ and $l_{y'}$ of the nanopillars.

Here, θ is the angle of nanopillar, $\varphi_{x'}$ and $\varphi_{y'}$ are the phase accumulated on the polarization component decomposed along the direction x' and y' respectively, which are determined by length $l_{x'}$ and $l_{y'}$. Thus, by properly designing the parameters $\{\theta, l_{x'}, l_{y'}\}$ of nanopillar at each (x_i, y_i), we can realize the desired phase configuration $\Phi^+_{\sigma_j}(x, y)$ and $\Phi^-_{\sigma_j}(x, y)$ for polarization $|\psi^+_{\sigma_j}\rangle$ and $|\psi^-_{\sigma_j}\rangle$, respectively.

The numerical simulation results of $\varphi_{x'}$ and $\varphi_{y'}$ are shown in Supplementary Fig. 4(e) and Supplementary Fig. 4(f) respectively, in which we set $l_{x'}$ and $l_{y'}$ from 100nm to 300nm with interval of 5nm. The corresponding transmittances are shown in Supplementary Fig. 4(c) and Supplementary Fig. 4(d) respectively. The choice of $\{\theta, l_{x'}, l_{y'}\}$ depends on the polarization $|\psi^+_{\sigma_j}\rangle$ and $|\psi^-_{\sigma_j}\rangle$ we want to separate, and we will discuss separately.

1. *Separation of linear polarizations*

For linear polarizations $|\psi^\pm_{\sigma_z}\rangle$ and $|\psi^\pm_{\sigma_x}\rangle$, we set θ of all nanopillars to be a constant. In this sense, phase configuration is determined by phase $\varphi_{x'}$ and $\varphi_{y'}$ in Supplementary Eq. (45). Specifically, to separate polarization $|H\rangle$ ($|\psi^+_{\sigma_z}\rangle$) and $|V\rangle$ ($|\psi^-_{\sigma_z}\rangle$), we set $\theta = 0°$ that leads to $x' = x$ and $y' = y$. According to Supplementary Eq. (45), the transformation of $|H\rangle$ and $|V\rangle$ after a single nanopillar is

$$U_{\text{pillar}} |H\rangle = e^{i\varphi_{x'}} |H\rangle, U_{\text{pillar}} |V\rangle = e^{i\varphi_{y'}} |V\rangle. \tag{46}$$

According to the value of $\Phi^\pm_{\sigma_z}(x_i, y_i)$ at position (x_i, y_i), we determine $l_{x'}$ and $l_{y'}$ by $\varphi_{x'} \approx \Phi^+_{\sigma_z}(x_i, y_i)$ (Supplementary Fig. 4(e)) and $\varphi_{y'} \approx \Phi^-_{\sigma_z}(x_i, y_i)$ (Supplementary Fig. 4(f)). Note that the choice of $(l_{x'}, l_{y'})$ is not unique, and the transmittances with corresponding $(l_{x'}, l_{y'})$ shown in Supplementary Fig. 4(c) and Supplementary Fig. 4(d) should be as high as possible.

To separate polarizations $|+\rangle$ ($|\psi^+_{\sigma_x}\rangle$) and $|-\rangle$ ($|\psi^-_{\sigma_x}\rangle$), we set $\theta = 45°$ for all nanopillars. According to Supplementary Eq. (45), the transformation of a single nanopillar is

$$U_{\text{pillar}} |+\rangle = e^{i\varphi_{x'}} |+\rangle, U_{\text{pillar}} |-\rangle = e^{i\varphi_{y'}} |-\rangle. \tag{47}$$

Similar to the situation of $\Phi^\pm_{\sigma_z}(x_i, y_i)$, the value of $(l_{x'}, l_{y'})$ is determined by $\varphi_{x'} \approx \Phi^+_{\sigma_x}(x_i, y_i)$ and $\varphi_{y'} \approx \Phi^-_{\sigma_x}(x_i, y_i)$ along with the consideration of high transmittance.

2. Separation of circular polarizations

The design to separate circular polarizations $|L\rangle$ ($|\psi^+_{\sigma_y}\rangle$) and $|R\rangle$ ($|\psi^-_{\sigma_y}\rangle$) is different with the situations of linear polarizations. To realize $\Phi^{\pm}_{\sigma_y}(x_i, y_i)$, $\varphi_{x'}$ and $\varphi_{y'}$ in Supplementary Eq. (45) should satisfy $|\varphi_{x'} - \varphi_{y'}| = \pi$ [8]. With this constraint, a single nanopillar transforms $|L\rangle$ and $|R\rangle$ by

$$U_{\text{pillar}}|L\rangle = e^{i(\varphi_{x'}+2\theta)}|R\rangle, U_{\text{pillar}}|R\rangle = e^{i(\varphi_{x'}-2\theta)}|L\rangle. \tag{48}$$

The values of θ and $\varphi_{x'}$ are determined by

$$\begin{cases} \theta = \dfrac{\Phi^+_{\sigma_y}(x_i, y_i) - \Phi^-_{\sigma_y}(x_i, y_i)}{4} \\ \varphi_{x'} = \dfrac{\Phi^+_{\sigma_y}(x_i, y_i) + \Phi^-_{\sigma_y}(x_i, y_i)}{2} \end{cases} \tag{49}$$

For the convince of fabrication, we choose nanopillars with 16 different $(l_{x'}, l_{y'})$ shown with red dots in Supplementary Fig. 4(c)-(f), and the parameters are shown in Table 3. For the phase $\Phi^{\pm}_{\sigma_y}(x_i, y_i)$ at position (x_i, y_i), we calculate θ and $\varphi_{x'}$ according to Supplementary Eq. (49), and choose the closest $\varphi_{x'}$ in Table 3 for fabrication.

$l_{x'}$ (nm)	$l_{y'}$ (nm)	$\varphi_{x'}$ / 2π	$\varphi_{y'}$ / 2π	$\mathbf{T}_{x'}$	$\mathbf{T}_{y'}$
110	150	0.1544	0.5924	0.9365	0.7640
120	155	0.2594	0.7413	0.8770	0.8245
120	160	0.2718	0.7972	0.8710	0.8567
120	165	0.2948	0.8546	0.8646	0.8837
120	170	0.3108	0.9024	0.8579	0.8803
125	165	0.4453	0.8751	0.8087	0.8857
125	170	0.4723	0.9251	0.8016	0.8618
125	175	0.4963	0.9698	0.7950	0.7676
150	110	0.5924	0.1544	0.7640	0.9365
155	120	0.7413	0.2594	0.8245	0.8770
160	120	0.7972	0.2718	0.8567	0.8710
165	120	0.8546	0.2948	0.8837	0.8646
165	125	0.8751	0.4453	0.8857	0.8087
170	120	0.9024	0.3108	0.8803	0.8579
170	125	0.9251	0.4723	0.8618	0.8016
175	125	0.9698	0.4963	0.7676	0.7950

Supplementary Table 3. Selected nanopillar sizes and corresponding phases and transmittances in the circularly polarized detection region, where $\mathbf{T}_{x'}$ and $\mathbf{T}_{y'}$ are transmission coefficient of lights with polarization along x' and y', respectively.

B. Numerical simulation of the designed metasurface

We simulate the performance of designed metasurface employing finite-difference time-domain (FDTD) method. Limited by computational memory space, we scale down the size of metasurface to 21μm×21μm and perform the simulation. The distribution of power intensity on the focal plane with input polarizations of $|H\rangle, |V\rangle, |+\rangle, |-\rangle, |R\rangle$ and $|L\rangle$ are shown in Supplementary Fig. 5(a)-(f). It can be seen that the six incident polarizations can be split and focused at the designed positions on the focal plane. As expected, for a specific polarization, the field intensity of its orthogonal polarization on the focal plane is almost zero, while the field intensity of the other two groups of states is half of the total field intensity. Here, the focused field intensity is defined as the total energy within a circle centered at the focal spot, with a radius of 1.5 times the full width at half-maximum (FWHM) [9]. Arbitrary polarization state can be represented by the Stokes vector formalized as $\boldsymbol{S} = [S_0, S_1, S_2, S_3]^T$. The elements S_i are defined by

$$\begin{aligned} S_0 &= I_H + I_V = I_+ + I_- = I_R + I_L, \\ S_1 &= I_H - I_V, \\ S_2 &= I_+ - I_-, \\ S_3 &= I_R - I_L, \end{aligned} \tag{50}$$

where I_p is the power intensity of polarization component $p \in [H, V, +, -, R, L]$. The results of normalized Stokes parameter $\boldsymbol{s} = [s_1, s_2, s_3]$ with

$$\begin{aligned} s_1 &= \frac{I_H - I_V}{I_H + I_V}, \\ s_2 &= \frac{I_+ - I_-}{I_+ + I_-}, \\ s_3 &= \frac{I_R - I_L}{I_R + I_L}, \end{aligned} \tag{51}$$

are shown in Supplementary Fig. 5(g)-(h).

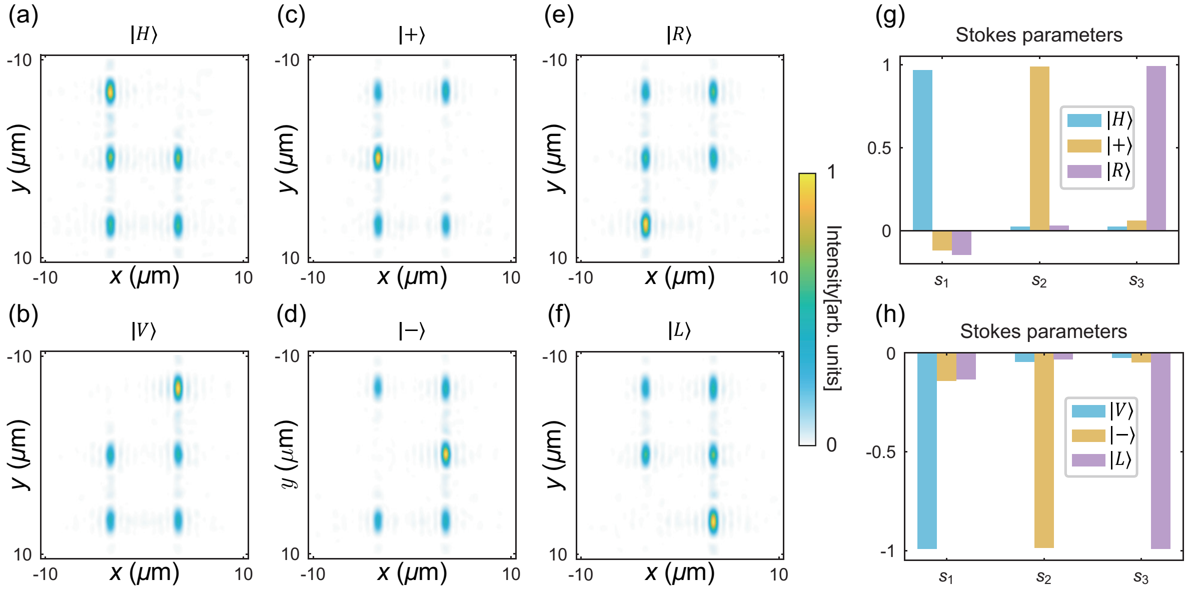

Supplementary Fig 5. **Numerical simulation of power intensity on the focal plane and reconstructed Stokes parameters. (a)-(f),** Spatial distribution of light intensity at the focal plane with input polarizations of$|H\rangle$,$|V\rangle$, $|+\rangle$, $|-\rangle$, $|R\rangle$ and $|L\rangle$. **(g)-(h),** The reconstructed Stokes parameters of the input polarizations of $|H\rangle$,$|V\rangle$, $|+\rangle$, $|-\rangle$, $|R\rangle$ and $|L\rangle$.

Compared to the ideal values, the average error of reconstructed Stokes parameters s_1, s_2 and s_3 are 0.097, 0.027 and 0.029 respectively, where the errors in $|H\rangle\,/\,|V\rangle$ basis is larger than that in $|+\rangle\,/\,|-\rangle$ and $|R\rangle\,/\,|L\rangle$ basis. This is mainly caused by the asymmetric response of region 1 with input polarization of $|H\rangle$ and $|V\rangle$. To verify this, we simulate the optical response of region 1 with input polarization of $|H\rangle$ and $|V\rangle$, respectively. The simulation is performed within range $x \in [-3\mu m, 3\mu m]$ and $y \in [-1\mu m, 1\mu m]$, which is scaling-down of region 1. As shown in Supplementary Fig. 6(a), the optical responses with input polarization of $|H\rangle$ and $|V\rangle$ are asymmetric with respect of y axis, leading to different transmit efficiency on focal plane. This is verified by simulation of distribution of power intensity on focal plane with input polarization of $|+\rangle = \frac{1}{\sqrt{2}}(|H\rangle + |V\rangle)$ as shown in Supplementary Fig. 6(d), where the output power intensities are unbalanced and introduces more errors in reconstruction of $s_1 = \frac{I_H - I_V}{I_H + I_V}$.

In contrast to region 1, region 2 ($|+\rangle/|-\rangle$ section) and region 3 ($|R\rangle/|L\rangle$ section) response their input polarization in symmetric manner. As shown in Supplementary Fig. 6(b) and (c), the distributions of response intensity with input polarization of $|+\rangle$ ($|R\rangle$) and $|-\rangle$ ($|L\rangle$) are symmetric with respect to $x = 0$, which consequently leads the balanced splitting of power intensity of input polarization $|H\rangle$ as shown in Supplementary Fig. 6(e) and (f). Therefore, the errors in reconstruction of $s_2 = \frac{I_+ - I_-}{I_+ + I_-}$ and $s_3 = \frac{I_R - I_L}{I_R + I_L}$ are smaller than that of s_1.

Discretization would also introduce optical loss. For example, different arrangements of nanopillars in three regions would introduce a "cut-off" in phase configuration on metasurface. We simulate the phase configuration on metasurface with input polarization of $|V\rangle$. As shown in Supplementary Fig. 7(a), there are two cut-off lines between three regions, which introduces undesired scattering and consequently increases the optical loss. However, this optical loss is small as most of the incident light is focused around the desired spot as shown in Supplementary Fig. 7(b) and (c), which is coupled into optical fibers in our experiment.

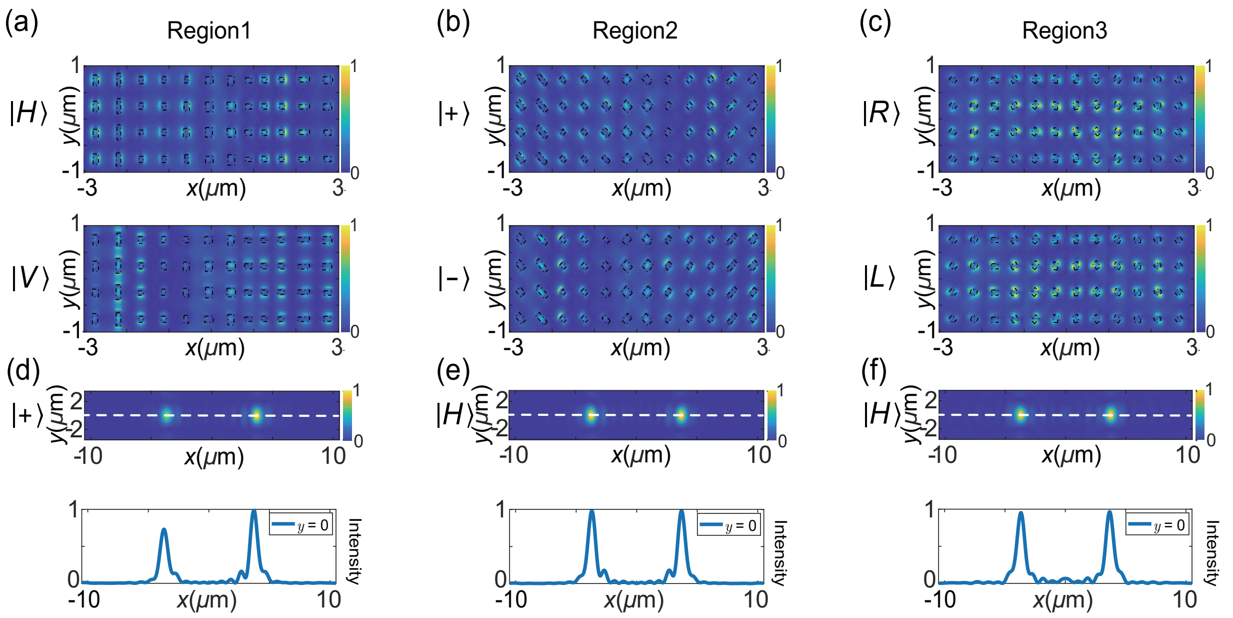

Supplementary Fig 6. Simulation of optical response of metasurface with different input polarizations. Optical response with input polarization of **(a),** $|H\rangle$ and $|V\rangle$, **(b),** $|+\rangle$ and $|-\rangle$, **(c),** $|R\rangle$ and $|L\rangle$. The distribution of power intensity on focal plane with input polarization of **(d),** $|+\rangle$, **(e),** $|H\rangle$ and **(f),** $|H\rangle$. The power intensity are represented in arbitray unit.

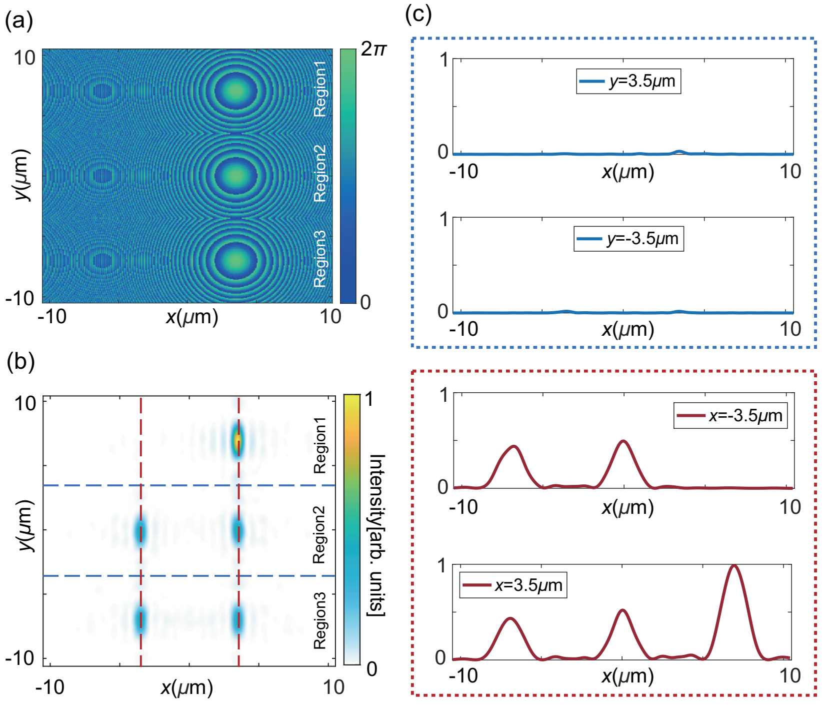

Supplementary Fig 7. Simulation of phase configuration and distribution of intensity on focal plane with input polarization of $|V\rangle$. (a), The phase distribution on metasurface. **(b),** The distribution of intensity on the focal plane. **(c),** The distribution of intensity along the dashed lines in **(b)**.

C. Experimental reconstruction of Stokes parameters of polarization with metasurface

The setup to reconstruct the Stokes parameters is shown in Supplementary Fig. 8. The wavelength of the incident light is 810nm followed by combination of a linear polarizer and a quarter-waveplate (QWP), which manipulates

the polarization of incident light. Then, the transmitted light focused on the focal plane is captured by a 20× objective lens (OL) and recorded on a CMOS image sensor. We test our metasurface with six input polarizations $p \in [H, V, +, -, R, L]$, and record the distribution of power intensity on the focal plane for each input. According to the power intensity, we calculate the normalized Stokes parameter $\boldsymbol{s} = [s_1, s_2, s_3]$, and the results are shown in Figure 1 in main text.

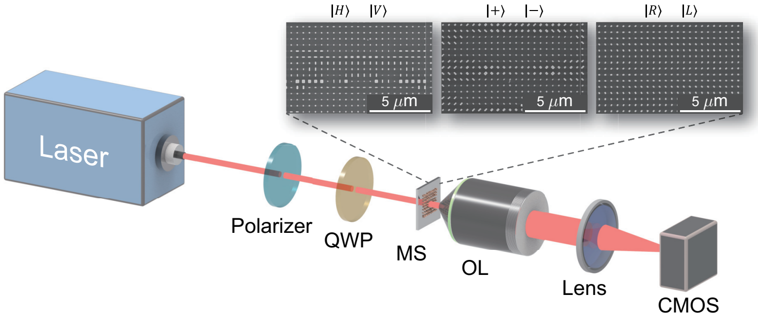

Supplementary Fig 8. Experimental setup to reconstruction of Stokes parameters of polarization with metasurface. HWP: half-wave plate. QWP: quarter-wave plate. MS: metasurface. OL: objective lens. CMOS: complementary metal-oxide-semiconductor.

D. Imperfections in metasurface

As shown in main text, the experimental results of reconstruction of Stokes parameters, shadow tomography estimation and MLE reconstruction conclude that the metasurface introduces measurement errors from 0.07 to 0.1. The errors are mainly attributed to the limitations in design of metasurface and the imperfections in fabrication of metasurface.

The key ingredient in the design of metasurface is to discretize the phase front. According to requirement of deflecting individual polarization to desired direction, we calculate a discrete phase accumulation of metasurface. Then, a periodic array of nanopillars is designed to realize such a discrete phase accumulation. However, the main limitation in such design is discretization itself, which inevitably introduces polarization measurement errors in our case. The discretization of phase front is an approximation of its continuous counterpart (bulk optics), which limits the accuracy of phase modulation. Consequently, the metasurface to deflect input polarization (for example $|H\rangle$) to desired direction cannot completely block its orthogonal polarization ($|V\rangle$) transmitting along the same direction, which introduces polarization measurement error. Particularly, to separate circular polarizations, i.e., $|R\rangle$ and $|L\rangle$, the cross-polarization effect has been employed in the design of metasurface, in which the conversion between two orthogonal circular polarizations ($|R\rangle \to |L\rangle, |L\rangle \to |R\rangle$) is firstly taken place on the metasurface. However, it is still challenging to realize complete conversion between $|R\rangle$ and $|L\rangle$ on metasurface. Specifically, the aspect ratio constraints of nanopillars, adjacent coupling between nanopillars as well as material absorption would reduce the efficiency of conversion, which consequently increases the errors in polarization measurement.

On the other hand, the metasurface to realize discrete phase modulation can be considered as a grating. For large bending angles (43° in our design), it inevitably deflects the incident polarization into other grating orders (undesired directions) [10].

Indeed, there are several schemes and techniques can improve the performance of metasurface.

High-order diffraction suppression. The high-order diffraction caused by the beam deflection can be suppressed by design of metasurface. For example, asymmetric grating profile [10] and nonperiodic metagrating designs [11] have been proposed to suppress high-order diffraction and deflect the light into a single desired order.

Multi-layer metasurface. Stacking multiple layers of metasurface with varied polarization filtering functionalities is able to enhance the accuracy of polarization control. For example, utilizing the double-layer chiral metasurface [12], the average measurement errors of Stokes parameters s_1, s_2, and s_3 were achieved at near-infrared wavelengths of 1.9%, 2.7% and 7.2%, respectively. The corresponding results in our work are 10.1%±0.5%, 8.6%±0.5% and 7.3%±0.5%.

Calibration. For general applications, the calibration process could eliminate errors introduced by inhomogeneity of the incident light on metasurface, including power intensity and incident angle [13]. Particularly, it has been proved that after calibration, the performance of metasurface-enabled polarimeter is comparable to that of bulk optics [14].

SUPPLEMENTARY NOTE 5: REALIZING PROJECTION ON $|\psi_l\rangle$ WITH EQUAL PROBABILITY

It is impossible to equally split input light into three regions due to the mode mismatch between incident light (Gaussian beam) and metasurface (square). Instead, we post select the photons passing through each region with equal probability. First, the beam waist of the input light is carefully adjusted to be $w_0 = \sqrt{2} \times 210$ μm with lens, which enables the maximal overlap between beam waist and metasurface (210 μm square). Then, we carefully locate the metasurface at the center of beam waist, which enables the equal probability of a single photon passing through region 1 ($|H\rangle / |V\rangle$ basis) and region 3 ($|R\rangle / |L\rangle$ basis), i.e., the count rates of collected photons passing through these two regions are the same. However, the single photon passes through region 2 ($|+\rangle / |-\rangle$ basis) with higher probability due to the nature of Gaussian distribution. In our experiment, we randomly discard the collected photons passing through region 2 to make the count rate equal to that of region 1 and region 3. Such experimental setting enables the equal probability of single photon passing through three regions.

SUPPLEMENTARY REFERENCES

[1] S. Chen, W. Yu, P. Zeng, and S. T. Flammia, PRX Quantum **2**, 030348 (2021).
[2] R. Penrose, Math. Proc. Cambridge Philos. Soc. **52**, 17 (1956).
[3] H. C. Nguyen, J. L. Bönsel, J. Steinberg, and O. Gühne, Phys. Rev. Lett. **129**, 220502 (2022).
[4] H.-Y. Huang, R. Kueng, and J. Preskill, Nat. Phys. **16**, 1050 (2020).
[5] J. Spall, IEEE Trans. Automat. Contr. **37**, 332 (1992).
[6] J. Spall, IEEE Trans. Aerosp. Electron. Syst. **34**, 817 (1998).
[7] J. Maryak and D. Chin, in *Proceedings of the 2001 American Control Conference. (Cat. No.01CH37148)*, Vol. 2 (2001) pp. 756–762 vol.2.
[8] S. Li, X. Li, G. Wang, S. Liu, L. Zhang, C. Zeng, L. Wang, Q. Sun, W. Zhao, and W. Zhang, Adv. Opt. Mater. **7**, 1801365 (2019).
[9] F. Balli, M. Sultan, S. K. Lami, and J. T. Hastings, Nat. Commun. **11**, 3892 (2020).
[10] D. Sell, J. Yang, S. Doshay, R. Yang, and J. A. Fan, Nano Lett. **17**, 3752 (2017).
[11] B. Born, S.-H. Lee, J.-H. Song, J. Y. Lee, W. Ko, and M. L. Brongersma, Nat. Commun. **14**, 5602 (2023).
[12] A. Basiri, X. Chen, J. Bai, P. Amrollahi, J. Carpenter, Z. Holman, C. Wang, and Y. Yao, Light: Sci. Appl. **8**, 78 (2019).
[13] S. Tang, T. Cai, H.-X. Xu, Q. He, S. Sun, and L. Zhou, Appl. Sci. **8** (2018), 10.3390/app8040555.
[14] L. W. Li, N. A. Rubin, M. Juhl, J.-S. Park, and F. Capasso, Appl. Opt. **62**, 1704 (2023).
